# Stakeholders’ awareness and perception towards graphic health warning, opportunities and challenges for tobacco control policy in Nepal: A qualitative study

**DOI:** 10.1371/journal.pgph.0004917

**Published:** 2025-07-10

**Authors:** Netra Lal Aryal, Binita Kumari Paudel, Babu Ram Pokhrel, Sushila Acharya, Saraswati Bhandari, Sheetal Bhandari, Manish Rajbanshi, Shalik Ram Dhital

**Affiliations:** 1 School of Health Sciences, Purbanchal University, Morang, Nepal; 2 Research Department, Lumbini Eye Institute, Bhairahawa, Nepal; 3 School of Public Health and Community Medicine, B.P. Koirala Institute of Health Sciences, Dharan, Nepal; 4 Central Department of Public Health, Institute of Medicine, Tribhuvan University, Kathmandu, Nepal; 5 Concern Center for Rural Youth, Kathmandu, Nepal; McGill University, CANADA

## Abstract

Despite the efforts of the Government of Nepal, including the World Health Organization (WHO) Framework Convention on Tobacco Control (FCTC), including 90% of health risk warnings on tobacco packets, the prevalence rate of tobacco consumption and its mortality and morbidity remain a challenge. This study aimed to explore awareness and perception towards Graphic Health Warning (GHWs), challenges, and opportunities for tobacco control policies among stakeholders in Galyang Municipality of Syjanga district, Nepal.This study employed a qualitative study design among 11 stakeholders of the Galyang municipality. Key Informant Interviews (KII) were conducted using the KII guideline. A purposive sampling technique was used to select study participants. The study adhered to the Consolidated Criteria for Reporting Qualitative Studies (COREQ) guideline. Thematic analysis was performed for data analysis.Participants, including Mayors, Ward Chairpersons, Health Officials, and community leaders, generally recognized GHWs as effective in raising awareness but noted their limited impact on altering smoking behavior, particularly among dependent smokers. While GHWs were seen as more effective than text warnings, many participants emphasized the need for stronger policy enforcement, including restrictions on tobacco sales and smoking in public places. Cleared, high-contrast images on cigarette packets, comprehensive public awareness programs, and active engagement of political leaders and stakeholders to strengthen tobacco control policies and implementation. This study found that GHWs raise awareness about the harms of tobacco, but their impact on behavior change, particularly among dependent smokers, remains limited. Key challenges include weak enforcement of tobacco control policies, regulatory gaps, and limited government engagement. A multi-sectoral approach with community leaders, political commitment, school-based intervention, stronger local governance, licensed tobacco shops, and enhanced GHWs is vital for effective tobacco control. Effective policy execution and community involvement were highlighted as critical to the success of tobacco control initiatives.

## Introduction

Tobacco consumption is a global public health challenge in the 21^st^ century and is one of the greatest preventable causes of premature deaths [1]. Tobacco is one of the major risk factors for Non-communicable diseases (NCDs) such as oral cancer, esophageal cancer, chronic obstructive pulmonary disease, and cardiovascular diseases [2]. Tobacco kills half of its users who don’t quit smoking in their lifetime, and contributes to poverty due to diverting spending income on basic needs such as food and shelter to tobacco products [3].

Globally, it was predicted that 8 million deaths are caused by tobacco each year, and around 80% of the world’s 1.3 billion tobacco users reside in Low- and Middle-Income Countries(LMICs), where tobacco control measures are often weaker and the health burden is greatest [4]. Tobacco consumption rates vary across countries, ranging from relatively high percentages, like in Nauru, Myanmar, and Serbia, to much lower rates in countries like Nigeria, Panama, and Ghana [5]. The rates vary significantly, indicating differing cultural, social, and economic factors influencing tobacco consumption worldwide [6]. Despite progress in tobacco control efforts, diverse patterns have been observed across countries [7]. Smoking indirectly or directly drives the increase in out-of-pocket payment for health care due to smokers spending their income on tobacco and smoking-related illnesses [8].

The 2019 STEPS (Step-wise approach to non-communicable disease risk factor surveillance) Survey conducted by the Nepal Health Research Council (NHRC) revealed that 28.9% of adults aged 15–69 currently use tobacco in some form, either smoked or smokeless [9]. This corresponds to approximately 3.8 million adults and represents a slight increase in the number of users, similarly, the NHRC’s study from 2016 to 2018 reported an overall tobacco use prevalence rate of 36.8% [9]. Tobacco use in Nepal is significantly higher than in other low-income countries, with 52.3% of men and 8.4% of women consuming tobacco [10]. In 2015, the World Health Organization (WHO) estimated 21.6% of Nepalese smoked, compared to 11.6% in low-income countries overall. Nepal’s tobacco use also exceeds most South Asian nations, except Bangladesh [10].

In response to control the tobacco epidemic in Nepal, health promotion activities are implemented for the Nepalese population by the National Health Education, Information and Communication Center (NHEICC) and other health units and volunteers with the technical support of the WHO [11]. In 2003, the Government of Nepal (GoN) joined the WHO Framework Convention on Tobacco Control (WHO-FCTC) and agreed to follow the convention [3]. The GoN introduced the Tobacco Product Control Act in 2011, ensuring legal provisions to reduce, control, and regulate the import, production, sale, distribution, and consumption of tobacco products [12].

The GoN became strict about tobacco advertisements in 2010, saying not to advertise any kind of tobacco-related information [12]. Besides, the government communicated to the tobacco companies that they couldn’t advertise anywhere else in 2006 and 2009 [3,12,13]. In 2011, the GoN decided that tobacco packs needed large warning labels that covered most of the package [12]. They even had different warnings for different tobacco products, all in the Nepali language. In Nepal, the Tobacco Product Control and Regulatory Directive 2014 mandated that health warnings must cover up to 90% of the front and back sides of tobacco product boxes and cartons [14,15].

Despite the implementation of GHW on tobacco packaging, there is a lack of understanding regarding their effectiveness in conveying health risk messages and influencing smoking behavior among adult smokers [4]. The NHEICC has already implemented a municipality-level campaign for the prevention and control of tobacco advertisements and its strict rule-out in 2021–22 [4]. However, the follow-up activities are yet to be implemented. While the GHW approach was introduced in Nepal to raise awareness, its effectiveness in influencing behavior change among smokers remains largely unexamined.

GHW has been found to help people understand the dangers of smoking, make health risks feel more serious, and encourage smokers to quit, while also helping stop young people from starting to smoke [11,14]. These warnings often cause strong emotional reactions, like fear or disgust, which makes them easier to remember and more likely to change behavior [3,8,16,17]. Still, putting GHWs in place can be difficult due to pushback from the tobacco industry, legal issues over branding, and delays from government or policy execution [2,11].

Hence, this study would provide additional evidence to improve the effectiveness of GHWs in raising awareness among people. Further, this study explores opportunities to improve challenging areas of tobacco control policies and programs, such as low enforcement of existing regulations, limited public awareness, inadequate cessation support services, and gaps in addressing emerging tobacco products. This study aimed to explore awareness and perception toward GHWs, challenges, and opportunities for tobacco control policies among stakeholders in Galyang Municipality of Syjanga district, Nepal.

This study is guided by two central research questions. The first seeks to examine the awareness and perceptions of GHWs among various stakeholders in Nepal, including local leaders, health professionals, school teachers, shopkeeper and smoker. The second research question explores the broader challenges and opportunities related to tobacco control policies in Nepal. This includes examining issues such as policy implementation gaps, enforcement barriers, public engagement, and multi-sectoral approach.

## Materials and methodology

### Ethics statement

Ethical approval was obtained from the Institutional Review Committee (IRC) of the School of Health Sciences, Purbanchal University, Nepal (Ref No 035–080/81). A letter of support was obtained from Galyang Municipality before data collection. The purpose of the study was clearly explained to the study participants. A written consent was taken from each participant. Participants were informed about the voluntary nature of their participation and their right to withdraw from the interview at any time. All information was kept confidential, and the data collected was used solely for research purposes.

### Study design

We conducted a qualitative study using Key Informant Interviews (KIIs) as the data collection method to explore awareness and perception toward GHW, challenges, and opportunities for tobacco control policies among stakeholders in Galyang Municipality of Nepal.

### Study setting

The study was conducted in Galyang Municipality of Syangja district, Gandaki Province. This municipality consists of a total of 11 wards. According to the STEPs Survey 2019, tobacco use amongst the population aged 15–69 years in Gandaki was 26.0% [18]. Nepal’s tobacco control is governed primarily by the Tobacco Product (Control and Regulation) Act, 2011. This law provides a comprehensive framework to regulate tobacco use, production, distribution, and promotion in Nepal. Galyang Municipality includes both urban and semi-urban areas, which may contribute to an increased risk of tobacco use due to easier access and changing lifestyle factors. Despite the implementation of GHW on tobacco products in Nepal, there has been no prior research specifically exploring the perceptions of GHW and their effectiveness in this region. Thus, this qualitative study will explore perceptions and challenges towards GHW and tobacco control policies among different stakeholders of Galyang Municipality.

### Study population

The study participants were elected leaders, School Teacher, Shopkeeper, Journalist, Ever-smoker, Health Section (HS) Chief, Hospital administrator, and Education, Youth, and Sports Section (EYSS) Chief of the Galyang Municipality.

The rationale behind selecting these specific participants is based on their direct influence and involvement in tobacco control at various levels, ranging from policy creation and enforcement to education and public health advocacy [12]. Elected leaders support policy advocacy, teachers raise awareness among students, shop owners ensure adherence to tobacco sales regulations, journalists inform the public, ever smokers offer personal perspectives, HS Chief oversee prevention programs, hospital administrators implement tobacco-free policies, and the EYSS Chief foster tobacco-free environments in schools and youth programs [12]. Together, their efforts are essential for effective tobacco control.

### Sample size and sampling techniques

This study included a total of 11 participants from diverse backgrounds for a KII for data collection. The purposive sampling technique was used to recruit participants. These participants were purposively chosen to capture insights from individuals who hold positions of responsibility and influence in tobacco control. These participants were selected for their expertise and active roles in sectors that are central to tobacco regulation, prevention, and cessation efforts. Their collective perspectives provide a comprehensive understanding of the multi-faceted approach required to combat tobacco use and its public health consequences.

### Data collection

The data was collected between February 15 to April 10, 2024. KII were carried out using the KII guideline to collect information for an in-depth understanding of perceptions and challenges towards GHW and tobacco control policies from diverse backgrounds.

Data collection was conducted using KII guidelines, and each interview took about 30–45 minutes. The KII guideline included domains such as awareness and perception, and the role of GHW, policy perspectives (including existing policies, challenges, and opportunities), as well as stakeholder recommendations and perspectives on collaboration.

KII tools were developed under the consultation of subject experts and an extensive literature review. The tool was pretested and finalized before collecting data from participants. This study followed Consolidated Criteria for Reporting Qualitative Studies (COREQ) guidelines to conduct, analyze, and report the findings of this study [19].

The Principal Investigator (NLA) was responsible for conducting interviews, and the researcher (SA) was responsible for note-taking and recording. The interviews were scheduled according to participant preferences, allowing them to engage in the interview process without external disturbance.

### Data management and analysis

All participants’ information was recorded using a tape recorder. The audio recordings and field notes from the interviews, conducted in Nepali, were transcribed and translated into English by SB and SA. The analysis process involved immersing fully in the dataset to develop an analytical framework and identify key themes, with cross-verification completed by MR and SRD.

This study followed Clark and Braun’s six-step approach to guide our thematic analysis [20]. An inductive thematic analysis approach was used to generate themes and codes. Then, Microsoft Excel software was used to conduct manual coding analysis. Two researchers independently developed codes and themes, which were then compared for similarities and differences. The analysis continued with the refinement and finalization of themes, ensuring clear definitions for each. Verbatim quotations from participants were included to support the emerging themes.

Key themes identified from the analysis included: perceptions of GHW messages, the role of GHW, challenges and opportunities in tobacco control policies, and strategies for effective implementation of GHW.

## Results

A total of 11 individuals participated in the study. (Table 1)

**Table 1:** Individual characteristics of the participants.

Participants	Age	Sex	Religion	Ethnicity	Education Level
Mayor	51	Male	Hindu	Brahmin	Master
Ward Chairperson	40	Male	Hindu	Brahmin	Bachelor
EYSS Chief	57	Male	Hindu	Brahmin	Master
HS Chief	36	Male	Hindu	Chhetri	Master
Hospital Administrator	54	Male	Buddhist	Janajati	Bachelor
Professor	57	Male	Hindu	Brahmin	Master
Principal	52	Female	Hindu	Brahmin	Secondary
School Teacher	48	Female	Hindu	Brahmin	Secondary
Journalist	50	Male	Hindu	Brahmin	Bachelor
Shopkeeper	26	Male	Hindu	Brahmin	Primary
Ever-smoker	78	Male	Hindu	Brahmin	Primary

The findings of the study were structured in the major three thematic areas of stakeholders’ perception towards the GHWs on tobacco control initiatives, roles of policy, its challenges, and opportunity in tobacco control initiatives, and key recommendations as a way forward for effective implementation of GHWs message in line with available policies related to tobacco control.

## Theme 1: Stakeholders’ Perception of GHW Message

### Participant: Mayor

The mayor stated that GHW on tobacco packets was more effective than text warnings. Also, he expressed that GHWs raise awareness, look frightening, increase health consciousness, and make people fearful, which helps reduce smoking to some extent.

*“Previously, there were no pictures on cigarette packets, but now the pictures are effective in controlling smoking, though not completely.”*

### Participant: Ward chairperson

The Ward chairperson expressed that GHWs are designed to raise awareness and discourage smoking, especially among non-smokers and new smokers. However, regular smokers often ignore them, leading to little effect on smoking habits.

*“Smokers who are addicted may not change because of pictures.”*

### Participant: EYSS Chief

The EYSS Chief member stated that text messages are not unappealing, and smokers often ignore the warnings and continue smoking, even though the graphics make them feel afraid.

*“Pictures on packets are ignored by smokers, who continue to smoke despite them. Informative messages should be displayed in public places.”*

### Participant: Hospital administrator

The hospital administrator stated that smokers perceive the severity of smoking but tend to ignore the graphics, while non-smokers are aware and less likely to initiate smoking. The GHWs are more effective than text warnings, with some smokers reducing their smoking due to GHW. Some smokers are reducing cigarette consumption or seeking cessation services prompted by GHW.

*“Previously, picture was not mentioned, due to an increase in NCD in Nepal, pictorial warning was introduced in tobacco products.”*

### Participant: Professor

The professors from the local campus expressed their view that GHWs have not significantly changed the smoking behavior of dependent smokers, indicating the need for additional measures and strategies beyond graphics. Although some smokers felt vulnerable to the risks, they made little effort to reduce the number of cigarettes they smoked.

“*Smoking is injurious to health, it damages lungs, mouth, and hands. Pictures are ineffective for addicted smokers”.*

### Participant: School principal

The school principal expressed that GHW should raise awareness and encourage some smokers to reduce their consumption. The school teacher perceived that the GHW has limited impact on changing smoking behavior, particularly among dependent smokers, due to ineffective policy implementation.

*“Pictures are effective and play a positive role in reducing and quitting smoking.”*

### Participant: Journalist

The journalist expressed that GHW raises awareness but has minimal effect on the behavior of dependent smokers, necessitating stronger measures against the tobacco trade.

*“Pictures alone do not create public awareness or drive change, as people remain dependent. Other programs, such as meditation, should be promoted.”*

### Participant: Shopkeeper

Shopkeeper mentioned that participants are aware of GHW on cigarette packets, but they largely disregard them, continuing to buy cigarettes freely. While new smokers may reduce their consumption, the graphics have little impact on the smoking behavior of dependent smokers.

### Participant: Ever-smoker

Ever-smokers expressed that GHW failed to discourage people from smoking as they ignored messages and continued to smoke freely in public places due to ineffective policy enforcement and execution.

*“People ignore the pictures and prefer to buy cigarettes without them.”*

## Theme 2: Role of GHW, challenges and opportunities of policy in tobacco control initiatives

### Participant: Mayor and ward chairperson

The Mayor expressed his view that tobacco control policies are incomplete, necessitating restrictions on the tobacco trade. There is a lack of policy enforcement in areas such as smoking prohibition in public places, sales to minors, and distribution of loose cigarettes. Smokers often disregard regulations, reducing policies to mere formalities without effective implementation. Thus, GHW should be promoted through active utilization of social media, mass media, and awareness programs on tobacco control.

*“Awareness and orientation programs should be conducted at the community level.”*(Mayor)

*“Graphics alone are not enough. Social media, along with awareness programs from provincial and local governments, should highlight the effects of smoking regularly in the community.”*(Ward Chaiperson)

### Participant: EYSS chief

The EYSS Chief reported that intentionally blurred images and smaller health warning texts on cigarette packets aim to boost trade, highlighting the need for robust policies and prohibitions in public places. Policy implementation remains inadequate, especially concerning smoking at bus parks, hotels, and restaurants. Minors continue to access and consume tobacco products, emphasizing the importance of establishing licensed tobacco shops.

*“Graphics warning has a positive role. Text messages are ineffective, but smokers viewed the pictures and understood the effects of smoking on their health.”*

*“Separate shops for cigarettes should be established, and sales should be prohibited to minors and pregnant women. Smoking should be banned in public places, and rules should be strictly enforced.”*

### Participant: HS chief

Smokers often disregard graphics due to nicotine dependency, exacerbated by unclear color contrasts on the packaging. Utilizing mass and social media can enhance awareness of tobacco control. Although policies are standardized but their implementation is weak, which highlights the need for an apex body to ensure enforcement. Local governments must monitor tobacco usage and enforce penalties for smoking in public areas.

*“An effective government structure should be established from the local to the federal level to implement the tobacco control policy. The policy should cover the entire process, from tobacco manufacturing to consumption and its health impacts.”*

### Paticipant: Hospital administrator

Some smokers are reducing cigarette consumption or seeking cessation services prompted by GHW. Therefore, policies on tobacco control should be revised and strictly enforced, urging individual compliance as reported by the administrator.

*“Policies are well formulated, but there is a lack of implementation. Smokers often send minors to buy cigarettes. Each individual should take responsibility for following the rules. Shopkeepers should not sell to minors or provide cigarette sticks.”*

### Participant: Professor and school principal

The professor reported that the prohibition of smoking in public places, increased tobacco taxation, and discouraging tobacco trade need to be enforced to reduce and control the prevalence of tobacco.

The school principal expressed that regular parental meetings and community awareness programs on smoking hazards should be incorporated into school curricula for the prevention and control of tobacco consumption.

*“Engagement should involve all sectors and stakeholders. I am playing a role in raising awareness among school parents during parent meetings. Every individual and community should be made aware of the importance of reducing and stopping smoking.”(S*chool Principal)

### Participant: Journalist

Journalist expressed that comprehensive awareness programs and job opportunities for unemployed people are needed to minimize the tobacco demand. He suggested that a ban on smoking in public places and limiting the tobacco trade can be strengthened in collaboration with other sectors.

### Participant: Shopkeeper

The shopkeeper stated that there is a high demand for loose tobacco sales and distribution, highlighting the need for policy monitoring and public awareness by the government.

*“People are demanding to buy loose cigarette packets, and if not provided, they will purchase them from another shop.”*

*“The government should effectively monitor the policy, and awareness programs should be conducted for the public.”*

## Recommendations for effective implementation of GHW

### Participant: Mayor and ward chairperson

The Mayor recommended organizing health promotion sessions and awareness programs on GHW with political support. Individuals should follow rules and regulations, and tobacco trade restrictions must be enforced. Collaboration among stakeholders, including tobacco production companies, development partners, policymakers, and political leaders, should be ensured to formulate effective policies and programs to control tobacco.

*“Collaboration is necessary, but not effectively utilized. Policy-making is a vital responsibility of political leaders, yet they are not giving sufficient importance to the issue.”* (Mayor)

*“After federalism, awareness programs should be conducted at the ward level. Smokers often ignore the pictures, but elected political leaders, as role models, can play a key role in spreading awareness*.” (Ward chairperson)

### Participant: EYSS chief

Cigarette packets should feature clear, high-contrast images depicting the adverse effects of smoking on different organs, accompanied by a visual narrative illustrating the progression of harm. Separate tobacco-selling shops should be established, and collaboration among stakeholders should be prioritized to address policy challenges and discourage smoking, as suggested by EYSS Chief.

*“Political leaders should develop a vision and set goals that prioritize the health and safety of the public. Policies should be created to discourage smoking, with public policies focused on protecting public health.”*

### Participant: HS chief

Ensuring clear and high-contrast pictorial warnings on cigarette packets, using both mass and social media for awareness campaigns, and conducting orientation programs are essential. According to the HS Chief of the municipality, collaboration with stakeholders, including tobacco companies, advocacy groups, and policymakers, through meetings, seminars, and focused group discussions, is essential for developing effective tobacco control policies.

*“Due to unclear colors and visuals, there is misinformation on the packet. Therefore, clear and contrasting pictures should be used on the packet of a tobacco product.”*

### Participant: Professor, school principal, and school teacher

They emphasized the importance of introducing anti-smoking education in school curricula, holding regular parental meetings, and organizing awareness programs at both school and community levels. They highlighted the need for stakeholder collaboration to reduce tobacco production and engage political leaders in awareness campaigns.

*“Smoking hazard-related messages should be included in the school curriculum. Schools can initiate rallies and community programs, where children educate their parents.”* (Professor)

*“Meetings, seminars, and demonstration programs should be initiated by political leaders. Political parties should focus on public health and coordinate with various stakeholders.”* (School Principal)

### Participant: Journalist

A journalist recommended that adding multiple pictures with additional stories on the risk and consequences of tobacco consumption can enhance the effectiveness of GHW. Genuine collaboration among stakeholders is essential, focusing on social impact rather than personal interests.

### Summary of perception towards GHW, policy gaps, and recommendations on tobacco control

This table summarizes perceptions towards GHW, policy gaps, and recommendations on tobacco control among participants. Participants with similar perceptions and recommendations were categorized in the same group. (Table 2)

**Table 2:** Summary of perception towards GHW, policy gaps, and recommendations on tobacco control among participants.

Informants	Perception	Policy gaps	Recommendations
Elected leaders (Mayor, Ward Chairperson)	GHW creates awareness through fear. However, smokers ignore health warnings on tobacco products.	Poor implementation and regulation of tobacco control policies and programs.	Multi-sectoral approach to control tobacco in Nepal.
Teacher (Professor, School principal, Teacher)	Tobacco companies produced blurred pictures, making the information unclear.	Tobacco-related information is not sufficient in the school curriculum.	Schools should integrate tobacco-related information into their curriculum to create awareness among students.
Shopkeeper	Dependent smokers ignore graphic warnings and buy loose cigarettes.	The government should effectively monitor the policy, and awareness programs should be conducted for the public.	Multiple images with storytelling should be used, and the government must regularly monitor and update policies.
Ever-smoker	GHW is ineffective in changing the smoking behavior among dependent smokers.	People tend to smoke freely in public places due to a lack of policy enforcement.	Tobacco production should be minimized.
HS Chief	People are aware of the severity of tobacco consumption, but smokers ignore graphic health warnings.	Despite standardized policies, weak implementation highlights an apex body to ensure enforcement.	Local governments should monitor tobacco use and enforce public smoking penalties.
Hospital Administrator	GHWs are more effective than text warnings.	Policies on tobacco control should be strictly enforced, urging individual compliance.	Clear and high-contrast graphic health warnings should be promoted by tobacco companies.
EYSS Chief	Tobacco companies intentionally use blurred images on cigarette packets to boost trade.	The establishment of licensed tobacco shops is important to reduce access to tobacco products to minors.	Inclusion of political leaders in awareness programs to control tobacco consumption.
Journalist	GHW creates awareness, however, it fails to change behavior towards smoking.	A ban on smoking in public places and restrictions on the tobacco trade should be enforced in collaboration with other sectors.	To increase the effectiveness of GHWs, multiple pictures with additional stories on the risks and consequences of tobacco consumption should be enhanced.

## Discussion

This study aimed to explore awareness and perception towards graphic health warnings, opportunities, and challenges for tobacco control policy among stakeholders of Galkot Municipality, Nepal.

Almost all the participants acknowledged the awareness-raising potential of GHWs, particularly in comparison to text-based warnings. The GHW message is the best way to create awareness on tobacco control and reduce non-communicable diseases, which is consistent with a previous study conducted in India [21], Lao PDR [16], and Nepal [11,17]. The study found that graphic health warnings (GHWs), showing clear images of the health effects of smoking, created fear and made both smokers and non-smokers more aware of health risks. Some stakeholders believed that some smokers tend to decrease the consumption of tobacco products and think about quitting tobacco because of the graphic health warnings [17]. However, many individuals ignore GHWs and smoke openly in public places in Nepal.

Participants recognized that GHWs are effective than text messages in raising awareness about the health effects of smoking. These findings were consistent with a previous study conducted by Sychareun et. al in 2015 and by Shrestha et.al in 2022 [16,17]. There was a consensus that GHWs alone were not enough to bring about change in smoking behavior, particularly among dependent smokers, and is supported by the review article conducted by Hammond et.al [2].

Stakeholders highlighted challenges in enforcing tobacco control policies, including implementing smoking bans in public places, preventing sales to minors, and restricting the sale of loose cigarettes. This finding is supported by a systematic review conducted on tobacco control policies [21], Korea [22], and China [23]. Policy enforcement was often weak, with regulations in place but not effectively put into practice in the country.

Nicotine dependency was cited as a major factor contributing to smokers’ disregard for GHWs. This study suggested comprehensive tobacco control and preventive strategies, including school-based tobacco control interventions, involvement of the community in awareness programs, strict regulation on tobacco advertisement, increased taxation on tobacco sales, and stricter enforcement measures to address gaps in tobacco trade regulations. Stakeholders proposed various strategies to enhance the effectiveness of GHWs and tobacco control policies, which are in line with the previous studies [17,24,25]. These included organizing health promotion sessions, utilizing mass and social media for awareness campaigns, establishing separate tobacco-selling shops, and integrating anti-smoking education into school curricula.

The findings highlight the importance of addressing the limitations of GHWs and strengthening tobacco control policies to combat smoking effectively. Recommendations include utilizing mass and social media platforms for targeted awareness campaigns, focusing on the health consequences of smoking and the effectiveness of GHWs. Collaboration among stakeholders is essential for effective enforcement, which is consistent with one of the studies conducted in western Nepal [17] and India [24]. Combine GHW with other tobacco control measures, such as increased taxation, public smoking bans, and anti-smoking education in schools, to create a holistic approach to reducing smoking prevalence, and genuine collaboration among stakeholders, including government agencies, health organizations, tobacco companies, and advocacy groups, to formulate and implement effective tobacco control policies, which is in line with the previous studies [18,21,22].

Like previous studies, this study acknowledges the importance of GHW in raising awareness about the health risks associated with smoking, as supported by the studies in Nepal [11], Lao PDR [16] and Myanmar [8]. This study found that all the participants perceived GHW as more effective than text-based warnings. Consistent with earlier findings, this study highlights challenges in effectively implementing tobacco control policies.

While previous studies recognize the limited consequences of GHW on smoking behavior, the current study suggested that GHW alone is insufficient to induce significant behavior change, particularly among dependent smokers. This study highlights additional recommendations for enhancing the effectiveness of GHW and tobacco control policies, such as a multi-sectoral approach, clear and contrast GHW integration of tobacco-related information in school curricula, prohibition of smoking at public places, community involvement in awareness programs, and is consistent with similar studies [26].

Furthermore, this study suggested the establishment of licensed tobacco-selling shops for tobacco supply. Both the GoN and WHO have documented that the GHW message is the best way of increasing awareness and reducing tobacco consumption, which is similar to this study [27]. This study suggests that GoN, including NHEICC, needs to coordinate and collaborate with the local level government for expanding the initiative of smoking-free municipalities in Nepal.

## Strengths and limitations

It is one of the first studies to explore GHW on tobacco products among different stakeholders. The findings would be useful to the NHEICC for the “Healthy Palika Initiative” program [26]. Participants were purposively selected to ensure the inclusion of perspectives from individuals occupying positions of authority and exerting significant influence in the field of tobacco control. While this study included a limited sample size of 11 participants, this research could be seen as a starting point and provide additional evidence to support future studies to deepen the understanding of contextual factors and policy implications. This study was based on a single municipality therefore, the findings cannot be generalized to other municipalities.

## Conclusion

This study highlighted the potential of GHW to raise awareness about the harms of smoking but doubted their effectiveness in influencing behavior, particularly among dependent smokers. This study identified key challenges such as weak enforcement of tobacco control policies and programs, gaps in regulating the tobacco trade and taxation, and the inactive role of government bodies in controlling tobacco consumption.

The study recommends a comprehensive approach to tobacco control, including integrating education on the health risks of tobacco use into school curricula, political commitment, leveraging social and mass media for awareness campaigns, and strengthening the enforcement of public smoking restrictions. It emphasizes the critical role of local government to monitor tobacco use and enforce penalties for violations in public spaces. Additionally, the study emphasizes the establishment of licensed tobacco shops and the use of high-contrast GHW on tobacco packaging to control tobacco consumption.

## Supporting information

S1 Data XXX.
(XLSX)
